# Sex Differences in General Knowledge: Meta-Analysis and New Data on the Contribution of School-Related Moderators among High-School Students

**DOI:** 10.1371/journal.pone.0110391

**Published:** 2014-10-27

**Authors:** Ulrich S. Tran, Agnes A. Hofer, Martin Voracek

**Affiliations:** Department of Basic Psychological Research and Research Methods, School of Psychology, University of Vienna, Vienna, Austria; University of Hertfordshire, United Kingdom

## Abstract

Research from various countries consistently reported an advantage of boys over girls in general knowledge and was also suggestive of some overall trends regarding specific domains of general knowledge that were speculated to stem from biologically differentiated interests. However, results were heterogeneous and, as of yet, had not been evaluated meta-analytically. Moreover, previous research drew on overly homogeneous high-school or undergraduate samples whose representativeness appears problematic; mostly, likely moderators, such as school type, student age or parental education, were also not directly investigated or controlled for. We provide a meta-analytical aggregation of available results regarding sex differences in general knowledge and present new data, investigating the psychometric properties of the General Knowledge Test (GKT), on which previous research primarily relied, and explored sex differences in a large and heterogeneous Austrian high-school student sample (*N* = 1088). The aggregated sex effect in general knowledge was of medium size in previous research, but differences in specific domains were heterogeneous across countries and only modest at best. Large sex differences in our data could be explained to a large part by school-related moderators (school type, school, student age, parental education) and selection processes. Boys had a remaining advantage over girls that was only small in size and that was consistent with the magnitude of sex differences in general intelligence. Analysis of the GKT yielded no evidence of biologically differentiated interests, but of a specific interest in the humanities among girls. In conclusion, previous research likely overestimated sex differences in general knowledge.

## Introduction

Sex differences in cognitive abilities and intelligence are a much investigated topic, with a host of studies documenting an advantage of women in verbal tasks and perceptual speed, but an advantage of men in visuospatial and complex numerical abilities [1]. Moreover, evidence points towards an advantage of men over women in fluid intelligence (Gf) [2]–[4], but also in crystallized intelligence (Gc) and general knowledge [5], [6].

Evidence from the standardization samples of information subtests of the Wechsler intelligence tests since 1958 consistently points towards an advantage of men in general knowledge [6], [7]. Yet, research points out that general knowledge is an intellectual ability sui generis (‘semantic long-term memory') that possibly needs to be regarded as another factor besides Gc and Gf, instead of being conceptualized as a verbal factor of Gc, and that may not be sufficiently explained by verbal ability, general intelligence, Gc or Gf alone [8]–[11].

Newer studies assessed general knowledge with a specific instrument, the General Knowledge Test (GKT) [9]. The original GKT consists of 216 items with an open response format that cover 19 different domains of knowledge. Its questions encompass “culturally valued knowledge, communicated by a range of non-specialist media” ([9], p. 859), like *Who discovered the double helix structure of DNA?* or *Which is the longest river in Asia?*, that are scored correct/incorrect, assigning half points to partly correct answers in some items. Irwing et al. [9] provided evidence that the 19 domains conform to a hierarchical second-order factor model, wherein six first-order factors (Current Affairs, Fashion, Family, Physical Health, Arts, and Science) load on the second-order factor general knowledge and account for the interrelations between the lower-order domains. Short forms, translations, and variants of the GKT were used in a number of further studies [10], [12]–[18].

A number of studies with the GKT addressed sex differences explicitly and provided converging evidence on sizable sex differences in general knowledge, and sometimes also with regard to underlying domains. There seems to be a specific male advantage in domains that are concerned with competition for status and power (Current Affairs, Physical Health), and a female advantage in domains that are concerned with nurturance and family (Family). This pattern was interpreted by some authors as suggestive of biological, i.e., evolutionarily developed and genetically predisposed, differences in interest between men and women, besides being determined by socialization and societal norms [6], [10], [17], [19]. Although there exists a systematic review on sex differences in general knowledge [19], there has been no attempt as of yet to aggregate the available evidence meta-analytically.

Previous studies on sex differences in general knowledge differed with regard to exact instrumentation, language and country, respondent age, socio-economic and educational background, and schooling. However, sex differences in intelligence and cognitive abilities may fluctuate from childhood to adolescence and adulthood [20], [21]. Moreover, boys show an overall greater variability in cognitive ability than girls on the population level [22], which may in part explain better cognitive achievements of boys and men in university or high-school samples, wherein the distribution of cognitive ability is likely truncated at or above the overall mean, leaving an excess of above-average boys and men, but excluding the excess of below-average boys and men. Furthermore, contextual and sociocultural factors need to be taken into account that, via cultural norms and stereotyping, may promote gender inequality, hinder girls' access to specific areas of education, lead to differential patterns of course taking in school and academia, or directly lead to underperformance in standardized tests in terms of self-fulfilling prophecies [23]–[26].

Studies that directly assessed sex differences in general knowledge and its domains relied on overly homogeneous samples whose representativeness seems problematic [27]. For example, undergraduates in [6] and [10] attended one and the same university. Eighty-three percent of undergraduates in [28] stemmed from one of two universities that were also in close geographical proximity (approximately 10 km apart). Participants in [18] stemmed from five German high schools of Marburg and Gießen, cities with roughly equally sized populations, located in the same county, approximately 33 km apart. All participants were from twelfth grade and hence similar in age. Samples reported in [19] may have been more heterogeneous. However, the provided information is insufficient to state so with certainty.

Differences in schooling and selection processes within educational systems are known to moderate sex differences in school achievement [29], [30]. Most bits of knowledge tested in the GKT have some affinity to contents typically taught in school. In agreement with this, general knowledge was found to predict school achievement [16] and to be higher in older students [15]. Moreover, parents' education may impact children's general knowledge, as it may also foster or limit school achievement [29], [31]. Lynn and Irwing [10] found only small effects regarding fathers' education. However, tests may have been overly conservative, owing to their homogeneous sample. Even though apparently more students were recruited from groups of lower socio-economic status at their study site (the University of Ulster) compared with other UK universities, nearly two thirds (63%) of fathers had the same level of education (secondary school to age 16), and another 14% had a quite similar (secondary school to age 18) educational background. Thus, fathers' education apparently did not vary much in this sample, limiting its potential effects on general knowledge.

The present study set out to provide a systematic account and aggregation of previously published evidence on sex differences in general knowledge and its domains and to investigate general knowledge among high-school students with new data, exploring specifically likely contributions of school-related moderators, such as school type, student age, and parental education. Participants were drawn from different types of high schools, different grades, and different geographical areas in Austria's largest county, Lower Austria. Effects of different school curricula were thus for the first time directly tested. Catchment area was systematically varied to attain a higher variability of socio-economic status and educational background of participants and parents than previous research and to obtain thus a more representative sample.

Moreover, Lynn et al. [18] translated the GKT into German, but based their item selection on the test performance of a sample of adults. Being intended for adolescents and young adults (i.e., high-school students and undergraduates), application of this item selection appears questionable. Furthermore, some GKT items seem typical for an Anglo-American background and may not be readily applicable in German-speaking countries. We therefore based our analyses on a new German form of the GKT, specifically selecting items appropriate for German-speaking high-school students. Moreover, the structural properties of the German GKT were thoroughly reassessed, using, unlike [9] and [18], methods that are specifically suited to its ordered categorical (dichotomous) item response format. There is evidence that the use of factor scores may reduce spuriously large sex differences in sum scores of tests of various cognitive abilities [32]. Therefore, analyses were based on factor scores in the present study.

## Meta-Analysis

### Method

#### Study inclusion criteria and literature search

**Figure 1:**
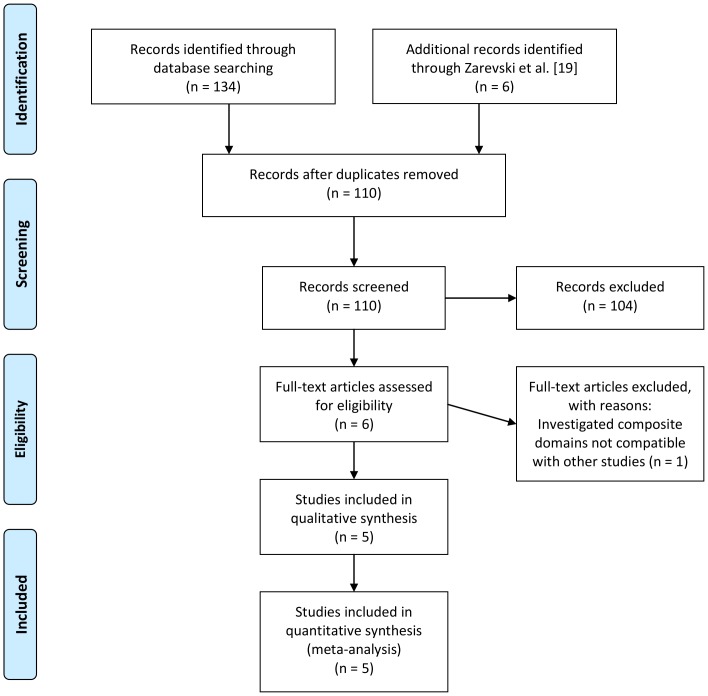
PRISMA flow diagram.

Published studies that reported sex differences in general knowledge as assessed with the GKT or instruments that could be readily likened to the GKT scheme, i.e., covered its lower-order domains or higher-order factors, were eligible for inclusion. Literature search drew on studies cited in [19] and on electronic databases (PubMed, PsycINFO, SCOPUS, Web of Science), using the search string (*general knowledge*) AND (*sex differences*). Six journal papers were identified for meta-analysis ([6], [10], [17]–[19], [28]; see Figure 1 for study flow). Results of [19] and [28] were not obtained with the GKT, but could be readily likened to its domains. Results of [17] were omitted because the (composite) domains investigated there (General Culture: composed of National and World History, and Arts; Natural and Social Sciences: composed of Natural and Social Sciences, Medicine, World's Religions and customs; Current Affairs: composed of Politics, Business, Technology, Sports and Entertainment) did not clearly fit into the overall scheme here. Deviating from the presentation of sum score differences in extant studies, [10] reported estimates that were derived with structural equation modeling (SEM) and also controlled for Gf; these values were used in the present analysis. Similarly, [28] reported on results controlling for Gf and Gc in regression analysis besides sum score differences. However, these results were not presented in a way that lent to meta-analytical aggregation; thus, only sum score differences reported in [28] were included here.

The finally included five primary studies report data from four countries (Croatia, Germany, UK, and USA); three studies reported sex differences among undergraduates, two among high-school students and high-school graduates, and one among pupils. The Zarevski et al. [19] study presented data from four samples (three pupil samples, one high-school sample); pupil samples were combined for analysis. Study details and findings are displayed in Table 1.

**Table 1:** Sex Differences in General Knowledge and its Domains in Previous Studies and Aggregated Effect Estimates.

Study	Lynn et al. [6]	Ackerman et al. [28]	Lynn & Irwing [10]	Lynn et al. [18]	Zarevski et al. [19]		
Sample	Undergraduates, University of Ulster	Undergraduates	Undergraduates, University of Ulster	High-school students	Pupils, 3 samples combined*	High-school graduates	
Country	Northern Ireland, UK	USA	Northern Ireland, UK	Germany	Croatia	Croatia	Aggregated mean effect
Mean or median age	20.4 years	19.0 years	20.5 years	18 years	15 years	18 years	[95% confidence interval]
Total *N* (% female)	635 (74%)	320 (53%)	1047 (57%)	302 (51%)	3156 (52%)	1174 (51%)	
*Current Affairs*	0.82	–	–	–	–	–	–
Politics	0.69	0.28		0.33	0.21	0.30	0.28 [0.23, 0.34]***
Finance	0.69	0.33	(0.48)	0.85	0.21	-	0.35 [0.29, 0.40]***
History	0.72	0.50^a^	–	0.62	0.20	0.08	0.26 [0.21, 0.31]***
Discovery	0.69	–	–	0.56	0.25	–	0.33 [0.26, 0.39]***
Geography	0.41	0.66	–	0.67	0.16	0.30	0.27 [0.21, 0.32]***
*Fashion*	-0.01	–	–	–	–	–	–
Fashion	-0.05	–	(-0.09)	0.03	-0.13	0.19	-0.05 [-0.10, 0.00]
Popular Music	-0.15	–	–	0.04^e^	-0.02	-0.21	-0.07 [-0.13, -0.02]*
Film	0.13	–	–	0.06	-0.24	0.08	-0.11 [-0.17, -0.06]***
*Family*	-0.46	–	–	–	–	–	–
Medicine	-0.32	–	(-0.24)	-0.20	-0.23	–	-0.24 [-0.29, -0.18]***
Cookery	-0.48	–	–	-0.50	-0.15	–	-0.22 [-0.28, -0.15]***
*Physical Health*	0.75	–	–	–	–	–	–
Biology	0.42	0.25	–	0.49	-0.15	-0.49	-0.11 [-0.17, -0.06]***
Games	0.54	–	(0.41)	0.82	–	–	0.51 [0.42, 0.60]***
Sport	0.84	–	–	1.12	0.31	0.28	0.39 [0.34, 0.45]***
*Arts*	0.31	–	–	–	–	–	–
Literature	0.49	0.10^b^	(0.21)	-0.09	-0.07	-0.12	0.01 [-0.03, 0.06]
Art	0.07	0.09	–	-0.16	-0.05	-0.24	-0.08 [-0.13, -0.02]**
Classical Music	0.08	0.20	–	-0.03	-0.28	-0.21	-0.19 [-0.24, -0.14]***
Jazz and Blues	0.46	–	–	–	–	–	–
*Science*	0.58	–	–	–	–	–	–
General Science	0.63	0.55^c^	(0.45)	0.55^f^	0.19	–	0.32 [0.27, 0.38]***
History of Science	0.33	–	–	–	–	–	–
**General knowledge**	0.51	0.68^d^	0.48 (0.67)	0.60	–	–	0.53 [0.45, 0.62]***^,g^

*Note.* Figures are standardized mean differences (Cohen's *d*) of sum scores. Positive values indicate that men/boys had higher scores than women/girls. Figures in parentheses were derived from structural equation modelling. * Weighted by inverse variance method. Mean of ^a^ ‘U. S. History’ and ‘Western Civilization,’ ^b^ ‘U. S. Literature’ and ‘World Literature,’ ^c^ ‘Chemistry’ and ‘Physics.’ ^d^ Composite of 19 domains of knowledge of which not all were commensurate to the domains of knowledge of the GKT. Composite scale of ^e^ ‘Popular Music’ and ‘Jazz and Blues,’ ^f^ ‘General Science’ and ‘History of Science.’ ^g^ Based on sum score estimates. * *p*<.05, ** *p*<.01, *** *p*<.001.

#### Data synthesis

A fixed-effect model was used for meta-analytical effect size aggregation, with Cohen's *d* as effect size [33]. Because of the known low power of Cochran's *Q* test to detect between-study effect heterogeneity when the number of studies is small [34], effect heterogeneity was assessed descriptively with the *I* ^2^ index. Heterogeneity was assumed small for *I* ^2^ = 25%, moderate for *I* ^2^ = 50%, and high for *I* ^2^ = 75% [34].

### Results and discussion

Sex differences in general knowledge were consistently of at least nearly medium size (i.e., | *d* |≥0.50), but differences in domains varied across studies in size but also in direction (Table 1). Results for Fashion, Popular Music, Film, Biology, Literature, Art, and Classical Music were inconsistent. Otherwise, boys and men excelled girls and women in all remaining domains across all studies, with the exception of Medicine and Cookery, where girls and women consistently excelled boys and men. SEM-based effect sizes [10] were mostly smaller than effect sizes obtained from sum scores in otherwise similar studies [6]. Sex differences were also smaller in samples of younger respondents [19].

The aggregated effect in general knowledge was of medium size, but effects were mostly only small (| *d |*<0.20) in the various domains (Table 1). Judging from the aggregated evidence, sex differences of at least medium size, favoring boys and men, were observable in Games, whereas small to medium effects were obtained in Sport, and in domains within Current Affairs and Science. Sex differences favoring girls and women were mostly negligible (| *d |≤*0.11); none were of even medium size. Effects that were at least small (| *d |*≈0.20) were obtained in Classical Music and in domains within Family. Domain-specific sex differences were thus mostly modest. Moreover, effect sizes in lower-order domains were considerable heterogeneous (*I* ^2^≥76%, except for Popular Music and Art, where *I* ^2^ = 0% and 19%, respectively), whereas with regard to general knowledge a homogeneous effect was observed (*I* ^2^ = 0%).

In total, the available evidence suggests a robust medium-sized advantage of boys and men over girls and women in general knowledge, whereas differences in lower-order domains were markedly smaller and inconsistent, and appeared to increase with age. On the content level, differences in general knowledge appeared to be mostly driven by domains within Current Affairs, Physical Health, and Science. Even though considerable effect heterogeneity was observed within these domains, effects were overall largest there and already observable at a relatively low age. Girls and women had an advantage in domains within Family that was already also observable at a lower age; however, the aggregated effect size was only small and effects were heterogeneous. In conclusion, the available evidence corroborates a male advantage in domains that are concerned with competition for status and power, and a female advantage in domains that are concerned with nurturance and family. However, there was also a considerable heterogeneity across different countries and studies with regard to these effects.

## Psychometric Analysis of the GKT and Contribution of School-Related Moderators on Sex Differences in General Knowledge

### Method

Ethical approval was obtained from the regional education authorities of Lower Austria (*Landesschulrat für Niederösterreich*), where the research was conducted. Ethic approval was then individually obtained from all participating schools as well, from the respective Head of School. Lastly, the parents of all participating students provided their written informed consent, as students were mostly underage and testing took place in a school context.

#### Construction of the German GKT

The GKT was translated, using forward and back-translation, into German and administered it to *N* = 21 (15 women) advanced and master thesis students of psychology at the University of Vienna. Instead of completing the test, raters rated item appropriateness on a 5-point scale (1 =  *very appropriate* to 5 =  *not appropriate*) with regard to whether each item represented “culturally valued knowledge but non-specialist information generally disseminated by the media” ([18], p. 1643) and whether each item was applicable to high school students (15–19 years) in Austria (neither too easy nor too difficult, suitable for German-speaking countries). Internal consistency (Cronbach's Alpha) of ratings was overall high (median α = .90; minimum α = .73 in Finance, maximum α = .96 in Art). Using a cutoff of 2.5, items with worse ratings were eliminated, resulting in the elimination of 53 items (25%). ‘Jazz and Blues’ was merged with ‘Popular Music’, yielding 18 domains covered by a total of 163 items. The number of items per domain in the German GKT may be gleaned from Table 2. The full German GKT may be obtained from the authors.

**Table 2:** Fit Statistics of Unidimensional Models in Domains.

Domain	Items	χ^2^	*df*	CFI	TLI	RMSEA [90%-CI]
Politics	10	68.13	35	.975	.968	.029 [.019,.040]
Finance	11	53.88	44	.990	.987	.014 [.000,.026]
History	9	36.25	27	.990	.986	.018 [.000,.031]
Discovery	11	45.58	44	.992	.991	.006 [.000,.021]
Geography	11	67.27	44	.981	.976	.022 [.010,.032]
Fashion	9	43.21	27	.966	.954	.023 [.008,.036]
Popular Music	10	132.77	35	.922	.900	.051 [.042,.060]
Film	8	45.21	20	.883	.836	.034 [.021,.047]
without Item 6	7	23.16	14	.949	.924	.025 [.000,.042]
Medicine	9	41.42	27	.959	.945	.022 [.005,.035]
Cookery	8	37.39	20	.979	.970	.028 [.013,.042]
Biology	9	35.58	27	.987	.982	.017 [.000,.031]
Games	6	15.82	9	.959	.932	.026 [.000,.047]
Sport	7	16.29	14	.999	.998	.012 [.000,.033]
Literature	8	30.22	20	.919	.886	.022 [.000,.037]
without Item 5	7	20.46	14	.953	.930	.021 [.000,.039]
Art	6	28.02	9	.919	.865	.044 [.026,.063]
without Item 4	5	10.34	5	.971	.942	.031 [.000,.059]
Classical Music	9	33.29	27	.985	.990	.015 [.000,.029]
General Science	10	55.89	35	.978	.972	.023 [.011,.035]
History of Science	11	99.29	44	.927	.909	.034 [.025,.043]

*Note.* CFI  =  comparative fit index, TLI  =  Tucker-Lewis index, RMSEA  =  root mean square error of approximation.

#### Participants and procedure

Based on the register of education authorities, 21 high schools were identified in the southern region of Lower Austria and contacted for the purpose of this study. Twelve (57%) schools consented to participate. Permissions to carry out the study in these schools were obtained from state education authorities, head teachers and parents. Testing took place in class rooms, with a time restriction of 45 minutes (in [9] the 216-item GKT was administered in 60 minute sessions). Participants also answered questions on socio-demographics and their parents' education. Altogether, 62 classes of grades 10 to 12 completed the assessment.

In total, 1088 students (743 girls, 345 boys) participated, aged 15–21 years (*M* = 16.6, *SD* = 0.9). Of these, 54.8% attended a high school with a focus on natural sciences (*Realgymnasium* in German; 5 schools), 18.4% (general) high schools (*Gymnasium*; 3 schools), 17.6% vocational schools (3 schools), and 9.3% schools of commerce (1 school). Overall, 54.2% attended grade 10, 40.1% grade 11, and 5.7% grade 12. Sex was imbalanced within school types: relatively more boys (69.9%) than girls (47.8%) attended high schools with a focus on the natural sciences, and fewer boys (5.2%) than girls (23.3%) attended vocational schools (χ^2^(3) = 66.45, *p*<.001). Students in schools of commerce were slightly older than all other students (*M* = 17.3 years vs. 17.0 years; *F*(3, 1079) = 5.79, *p* = .001; post hoc Tukey tests: *p*s≤.026).

With regard to living and catchment area, 29.0% of the students stemmed from communities with 5000+ residents, 53.0% from communities with 1000–5000 residents, and 16.3% from communities with less than 1000 residents; 1.7% provided no information. Of respondents' fathers and mothers, respectively, 46.0% and 47.9% had completed lower secondary school, 24.5% and 29.0% had completed upper secondary school, and 26.8% and 22.1% had a university diploma or a similar degree (2.7% and 1.1%, respectively, provided no information). Fathers' and mothers' educational background was fairly concordant (weighted kappa  = .53, *p*<.001).

### Analysis

#### Structure of the German GKT

Answers on the German GKT were scored 1 (*correct*) and 0 (*incorrect*). Partly correct answers (e.g., responding to *What are the chemical constituents of water?* solely ‘hydrogen’) were assigned half points. Unidimensionality of domains was investigated with confirmatory factor analysis, using Mplus 6.11 [35] and its weighted least square mean- and variance-adjusted (WLSMV) estimation option which is suited for ordered categorical variables and is based on the items' polychoric correlation matrix. Items were excluded where necessary to improve model fit.

In a second step, the higher-order factor model of [6] was fit onto the domain factor scores, using a robust maximum likelihood estimator (MLR) to guard against non-normality of data and to arrive at robust indices of model fit. As this model had no good fit on our data, and modification indices indicated a much more complicated loading pattern than originally assumed, a modified approach was chosen instead. In order to deal with the apparent cross-loadings of domains on first-order factors, exploratory structural equation modeling (ESEM [36]) was used, which estimates cross-loadings freely as in exploratory factor analysis, but also derives indices of model fit as in confirmatory factor analysis and structural equation modeling. ESEM was successfully utilized in previous research dealing with similar problems (e.g., [37]).

As of yet, it is technically not possible to fit a higher-order ESEM. Hence, analyses on the doubly-tiered hierarchical structure of the GKT were performed in two steps: first, with regard to domains and first-order factors, using domain factor scores; second, with regard to first-order factors and second-order general knowledge, using first-order factor scores. With regard to the former analysis, varying numbers of first-order factors were examined, selecting (1) the most parsimonious model (fewest factors) that had (2) a good model fit, (3) good interpretability, and (4) high factor determinacies (i.e., high correlations of factor score estimates with underlying factors).

Model fit was checked with CFI and TLI (comparative fit index, Tucker-Lewis index; acceptable fit: ≥.90, good fit: ≥.95), RMSEA (root mean square error of approximation; acceptable fit: <.08, good fit: <.06), and SRMR for MLR analyses (standardised root mean residual; acceptable fit: <.11, good fit: <.08) [38]. ESEM estimates a large number of parameters which may spuriously inflate the RMSEA [37] that penalizes for model complexity. This is similarly the case with the TLI. Hence, in ESEM analyses, model fit was primarily interpreted with regard to CFI values.

#### Sex differences and contribution of school-related moderators

Magnitude (Cohen's *d*) and significance of sex differences were assessed for domains, first-order factors, and second-order general knowledge with generalized linear models (GLM), using factor scores and investigating moderating effects of school type, school, parental education, and student age. Effect sizes were derived from GLM parameter estimates, presenting both naïve and unadjusted effect sizes (examining only sex in the GLM), and effect sizes adjusted for moderators (using the full GLM as outlined above). For first-order factors and second-order general knowledge, the dependent variable was modelled with a normal distribution in the GLM. For domain factor scores, Gamma distributions were used to account for observed skewness. The identity function was used as link function, the covariance matrix was estimated via a robust sandwich estimator. The GLM allowed to model heterogeneous variances (higher variance in boys) and the clustered nature of the data: students stemmed from different schools within the same school type. In the GLM, school was modelled as a nested factor within school type to account for the variability between schools and to safeguard against Type I errors due to cluster sampling.

A full (saturated) model was investigated with the GLM in order to avoid biased estimates of effect size [39]. However, instead of a main effect of age, the interaction of age by school type was tested in the GLM, to assess differences in years of schooling (age) between school types. Effects of fathers' and mothers' education were tested independently in separate GLMs. Post hoc comparisons were carried out utilizing sequential Bonferroni corrections.

Moreover, we also tested the association of parental education with school type (separate chi-square tests for mothers and fathers, and separately for boys and girls), to assess the overall contribution of parental education on their children's educational career. Significance was set to *p*<.05 (two-tailed).

### Results and discussion

#### Unidimensionality of domains

Unidimensional factor models had an adequate fit in all domains, with the exception of Film, Art, and Literature (Table 2). In Literature, Item 3 (*Who wrote ‘Doctor Zhivago’?*) had to be excluded beforehand, as no respondent knew the right answer. To improve model fit, Item 6 in Film (*Who played the leading male part in ‘Titanic’?*), Item 4 in Art (*Who painted the ‘Mona Lisa’?*), and Item 5 in Literature (*Who wrote ‘Politeia’?*) had to be removed as well. Compared to the other items, knowledge on these items was probably overly influenced by popularity effects (Film), pervasiveness in the mass media (Art), or in not being a prototypical example of its subject (Literature). In all resulting scales unidimensional models had at least an acceptable fit, most had a good fit.

#### Structure and reliability of the GKT

The hierarchical model of [6] had no good fit on our data (Table 3). Modification indices indicated that a large number of domains should be allowed to cross-load on other factors besides their designated factor. ESEM analyses with six, five, four, and three factors (Table 3) suggested that a 4-factor model fitted the data best. In the 6-factor model, one factor had no, another only one, significant loading. In the 5-factor model, two factors had factor determinacies <.80, whereas in the 4-factor model only factor 4 had a factor determinacy that was only slightly below.80 (Table 4). Moreover, the overall fit of the 4-factor model could be considered good and was considerably better than that of a 3-factor model (Table 3). The seemingly low loadings in factor 4 need to be interpreted in context (Table 4): Loadings of Games, Literature, Arts, and Classical Music on factor 4 were lower by at most 37% (Literature) to as few as 15% (Arts) compared to the highest loadings of these domains on the other factors. Moreover, the negative loading of Sport on factor 4 was higher than its positive loading on factor 1 (.52). There was no indication for a need to model correlated residuals. Therefore, the 4-factor solution was kept as the final model as it appeared most parsimonious.

**Table 3:** Fit Statistics of the Higher-Order Factor Models.

Model	χ^2^	*df*	CFI	TLI	RMSEA [90%-CI]	SRMR
Confirmatory hierarchical factor model of [6]	985.81	128	.862	.836	.078 [.074,.083]	.059
6-factor ESEM	98.64	60	.994	.984	.024 [.015,.033]	.010
5-factor ESEM	137.86	73	.990	.978	.029 [.021,.036]	.014
**4-factor ESEM**	**229.12**	**87**	**.977**	**.960**	**.039 [.033,.045]**	**.019**
3-factor ESEM	516.58	102	.934	.900	.061 [.056,.066]	.032

*Note*. CFI  =  comparative fit index, TLI  =  Tucker-Lewis index, RMSEA  =  root mean square error of approximation, SRMR  =  standardized root mean residual. The bold printed model was retained for further analysis.

**Table 4:** Loadings in the 4-Factor ESEM.

	GEOMIN-rotated factors			
Domains	1	2	3	4
*Current Affairs*				
Politics	**.85**	.00	−.09	−.00
Finance	**.60**	**.12**	**.19**	**−.16**
History	**.79**	**−.19**	.01	.09
Discovery	**.47**	−.01	**.20**	−.08
Geography	**.58**	−.05	**.15**	−.07
*Fashion*				
Fashion	.02	**.70**	.01	.01
Popular Music	.04	**.75**	.02	**−.10**
Film	**.35**	**.42**	−.13	−.01
*Family*				
Medicine	.09	**.21**	**.37**	**.22**
Cookery	−.06	**.43**	**.35**	.11
*Physical Health*				
Biology	.00	.08	**.69**	−.02
Games	.03	−.01	**.52**	**−.38**
Sport	**.52**	.06	.01	**−.55**
*Arts*				
Literature	**.30**	**.18**	**.23**	**.19**
Art	**.40**	**.15**	.01	**.34**
Classical Music	**.38**	.07	.01	**.26**
*Science*				
General Science	.22	**−.15**	**.64**	−.00
History of Science	**.48**	−.02	**.18**	**.22**
Factor determinacies	.95	.89	.88	.78

*Note*. All bold printed loadings were significant at *p*<.01. Underlined loadings indicate where domains loaded highest positively.

Factor 1 subsumed with highest loadings domains within Current Affairs and Art, but also Sport and History of Science (Table 4). Factor 2 subsumed with highest loadings domains within Fashion and Cookery, and factor 3 Medicine, Biology, Games, and General Science. Factors 1 to 3 incorporated a larger number of cross-loadings, some of them also negative, whereas factor 4 appeared special as it contained exceptionally large negative cross-loadings of Games and Sport. Medicine, Literature, Art, Classical Music, and History of Science all had positive cross-loadings on this factor. Moreover, factors 1 to 3 were positively inter-correlated (factor 1 with factor 2: *r* = .65, *p*<.001; factor 1 with factor 3: *r* = .40, *p*<.001; factor 2 with factor 3: *r* = .19, *p*<.001), but factor 4 had no associations of with any of the other factors (*r*s  = .03,.11, and.05 with factors 1 to 3, *p*s ≥.247). In a further ESEM analysis with the four first-order factors, factors 1 to 3 had large and significant loadings (.97,.78,.47, *p*s <.001) on second-order general knowledge, but not factor 4 (loading  = .04, *p* = .321). In this analysis, the variance of factor 1 had to be constrained to be positive to attain convergence. Fit of this 1-factor ESEM was good with regard to CFI, χ^2^(2) = 51.17, p<.001, CFI  = .957, TLI  = .871, RMSEA  = .150, 90% confidence interval  =  [.116,.187]. Factor determinacy of second-order general knowledge was also high,.97.

With regard to interpretation, factors 1 to 3 appeared components of a common positive manifold of general knowledge. According to its high second-order factor loading, factor 1 was virtually identical to general knowledge itself. Factor 2 and 3 appeared to encompass more specific areas of knowledge, i.e., aspects of what could be termed ‘recreational lifestyle’ (factor 2) and the natural and the life sciences (factor 3). Factor 4 seemingly lay outside this manifold. Yet, it contained cross-loadings of domains that were themselves components of the manifold. However, positive and negative effects apparently cancelled each other out, resulting in no overall association of factor 4 with second-order general knowledge. With regard to its contents, factor 4 appeared to encompass increased knowledge of the arts, Medicine, and History of Science, reminiscent of a specialisation in the humanities, but accompanied by a specific lack of knowledge especially in Games and Sport.

Reliability indices of sum scores were excellent for the total GKT, but unacceptably low (α<.60) in some domains (Table 5). However, given that domains were unidimensional, low reliabilities obviously stemmed from the dichotomous item format and the fact that there were only few items within some domains. Moreover, Cronbach's Alphas on the domain level in our study broadly matched previous figures [18].

**Table 5:** Scale Reliabilities, Sum Score Means and Standard Deviations, and Sex Differences in Factor Scores, Unadjusted and Adjusted for Moderators.

		Sum scores		Sex differences (Cohen *d*) in factor scores	
Domain	α	Boys	Girls	Unadj.	Adj.
*Current Affairs*					
Politics	.64	2.63 (1.78)	1.32 (1.30)	0.83***	0.15*
Finance	.60	3.61 (1.75)	1.99 (1.51)	1.05***	0.17*
History	.63	2.13 (1.87)	0.82 (1.11)	0.85***	−0.01
Discovery	.37	2.50 (1.19)	1.73 (0.92)	0.71***	0.11*
Geography	.66	2.52 (2.04)	1.34 (1.41)	0.69***	0.14*
Sport	.68	2.85 (2.01)	1.17 (1.10)	1.00***	0.19**
*Fashion*					
Fashion	.51	2.06 (1.36)	2.36 (1.59)	−0.36***	−0.11
Popular Music	.63	2.90 (1.86)	3.10 (1.74)	−0.10	−0.02
Film	.36	0.58 (0.89)	0.48 (0.72)	0.10	−0.01
*Family*					
Medicine	.41	3.14 (1.45)	3.27 (1.38)	−0.06	−0.01
Cookery	.58	3.06 (1.48)	3.30 (1.62)	−0.15*	−0.07
*Physical Health*					
Biology	.56	5.61 (1.66)	4.48 (1.82)	0.59***	0.15*
Games	.44	3.10 (1.33)	2.06 (1.28)	0.93***	0.05
*Arts*					
Literature	.34	1.12 (0.71)	0.94 (0.81)	0.26***	0.06
Art	.36	0.66 (0.77)	0.61 (0.85)	0.02	0.23***
Classical Music	.48	1.10 (0.93)	1.04 (0.91)	0.06	0.00
*Science*					
General Science	.60	5.44 (1.87)	3.72 (1.76)	0.96***	0.13*
History of Science	.45	2.12 (1.48)	1.52 (1.12)	0.42***	0.00
*First-order factors*					
Factor 1 (general knowledge)				1.03***	0.16*
Factor 2 (lifestyle)				0.94***	0.13
Factor 3 (sciences)				−0.27***	−0.05
Factor 4 (humanities)				−0.81***	−0.16*
Second-order general knowledge	.92	47.16 (15.02)	35.25 (13.41)	1.02***	0.16*

*Note*. α =  Cronbach's Alpha; Unadj./Adj.  =  unadjusted/adjusted for moderators in GLM analyses. Positive effect sizes signify an advantage of boys over girls. * *p*<.05, ** *p*<.01, *** *p*<.001.

#### Sex differences and contribution of moderators

In naïve estimation of sex differences, a large difference between boys and girls emerged in second-order general knowledge (Table 5). Even though effect size estimation was based on factors scores, we provide summary statistics on sum scores in Table 5 to enable direct comparisons with previous research. In first-order factors, the largest difference, favouring boys, was found in factor 1, closely followed by factor 2. However, differences were in favour of girls in factors 3 and 4; effect size was small in the former, but large in the latter. Differences in domain scores were mostly similar to differences in respective first-order factors. In sum scores, boys had a larger score variance in total general knowledge and most domains, with exceptions in Fashion, Family, Cookery, Physical Health, Biology, Literature, and Art, where girls had a larger score variance (Table 5). A similar pattern was observable in factor scores (not shown for brevity). Compared with [18] our results appeared to be on the large side on the domain level (+0.13*d* on average). Yet, a strong concordance was observable with regard to the relative magnitude of effect sizes across the different domains.

Adjusting for moderators, sex differences were greatly diminished in size. Overall, mothers' education had a stronger impact on general knowledge than fathers'; hence, we report here on results of GLMs incorporating only mothers' education (*N* = 1071 due to partially missing data). Table 6 displays effect tests with regard to investigated factors and interactions for first-order factors and second-order general knowledge. For brevity, only results on these higher-order factors, but not on domains, are detailed here. With regard to factor 1 and second-order general knowledge, we present details only for the latter, as findings were similar for both. Effects of sex robustly emerged in the analyses; however, they were systematically qualified by a number of interactions.

**Table 6:** Effect Tests in the GLMs.

	First-order factors				Second-order
	1	2	3	4	general knowledge
Sex	109.41(1)***	74.65(1)***	49.36(1)***	111.59(1)***	101.81(1)***
School type	18.83(3)***	6.26(3)	9.08(3)*	2.14(3)	17.90(3)***
School^a^	140.87(8)***	35.50(8)***	60.81(8)***	91.87(8)***	129.95(8)***
Mothers' education	2.41(2)	1.32(2)	6.77(2)*	3.09(2)	2.26(2)
Sex × school type	38.92(3)***	15.63(3)***	21.07(3)***	25.62(3)***	38.02(3)***
Sex × school^a^	88.69(8)***	27.50(8)***	46.84(8)***	54.87(8)***	97.48(8)***
Sex × mothers' education	2.16(2)	0.10(2)	7.22(2)*	1.10(2)	2.10(2)
School type × mothers' education	39.41(6)***	18.21(6)**	23.40(6)***	10.68(6)	37.63(6)***
School^a^ × mothers' education	17.52(16)	23.30(16)	16.99(16)	26.79(16)*	17.37(16)
Sex × school type × mothers' education	7.24(5)	2.27(5)	10.39(5)	9.15(5)	7.09(5)
Sex × school^a^ × mothers' education	28.40(12)**	24.99(12)*	15.51(12)	11.27(12)	27.60(12)**
School type × age	49.97(4)***	10.15(4)*	21.05(4)***	2.95(4)	44.83(4)***

*Note*. Entries are Wald χ^2^ tests with degrees of freedom in parentheses. ^a^ Nested within school type. * *p*<.05, ** *p*<.01, *** *p*<.001.

There was a main effect of school type on general knowledge, but not on first-order factors 2 to 4 (the effect on factor 3 was only small and negligible in size). In marginal means, general knowledge was highest in high schools, followed by high schools with a focus on the natural sciences, schools of commerce, and vocational schools (*d* = −0.35, −0.49, and −0.78, comparing the other school types, in the same order, to high schools; all pairwise comparisons were significant at an overall *p*<.05, except for the last two types of school, *p* = .129). Schools themselves impacted all first-order and the second-order factor significantly, indicating heterogeneity of individual schools that may stem from differences in catchment area, but also from differences between individual schools of the same type. Mothers' education exerted no significant main effect on any of the five dependent variables.

School type and individual schools qualified systematically, via first-order interactions, sex differences in all dependent variables. In marginal means, boys had higher factor scores in general knowledge than girls in all school types (largest difference in high schools, *d*s ranging from 0.52 to 1.11, *p*s ≤.016), but vocational schools (*d* = 0.10, *p* = .681), and higher scores in factor 2 in both types of high school (larger in high schools, *d* = 0.99, than in high schools with a focus on the natural sciences, *d* = 0.72, *p*s <.001), but not in vocational schools (*d* = −0.15, *p* = .559) or schools of commerce (*d* = 0.35, *p* = .103). In comparison, girls had higher scores than boys in factor 3 in all school types (largest difference in vocational schools, *d*s ranging from 0.34 to 0.64, *p*s ≤.034), but schools of commerce (*d* = 0.07, *p* = .729), and higher scores in factor 4 in all school types (largest difference in high schools, *d*s ranging from 0.61 to 1.40, *p*s ≤.004), but vocational schools (*d* = 0.27, *p* = .227). There was again systematic heterogeneity with regard to these effects on the level of individual schools.

Mothers' education impacted all dependent variables, except factor 4, via a first-order interaction with school type, altering the rank order of school types dependent on mothers' education, and with regard to general knowledge and factor 2 via a second-order interaction with sex and school. Factor scores were significantly higher in high schools than in all other school types, when mothers had completed upper secondary education, but, otherwise, differences were mostly significant only with regard to school of commerce (ranking last), when mothers had completed lower secondary education, and with regard to vocational schools (ranking last), when mothers had a university diploma.

Lastly, age affected general knowledge, and factors 2 and 3, dependent on school type. General knowledge increased systematically in high schools (*B* = 0.18, 95% confidence interval  =  [0.04, 0.33], *p* = .015) and vocational schools (*B* = 0.25 [0.17, 0.34], *p*<.001), but not in the other two school types (*p*s ≥.077). Factor 2 increased significantly only in vocational schools (*B* = 0.21 [0.06, 0.34], *p* = .004; other *p*s ≥.296). Factor 3 increased significantly in high schools with a focus on the natural sciences (*B* = 0.09 [0.01, 0.16], *p* = .025) and vocational schools (*B* = 0.25 [0.12, 0.37], *p*<.001; other *p*s ≥.232). Factor 4 remained unaffected by age.

School type was strongly dependent on mothers' and fathers' education. Associations were stronger for girls (mother: χ^2^(6) = 37.80, *p*<.001; father: χ^2^(6) = 44.69, *p*<.001) than for boys (mother: χ^2^(6) = 17.59, *p* = .007; father: χ^2^(6) = 24.23, *p*<.001). Fewer girls (13.8% vs. 25.8% with regard to mothers' education; 12.7% vs. 26.0% with regard to fathers' education) attended high schools, and more (13.6% vs. 4.7%, and 13.8% vs. 5.0%, respectively) schools of commerce, when parents had completed only lower secondary school, compared to higher educational levels. Vice versa, more girls (30.9% vs. 16.8%, and 26.9% vs. 16.9%, respectively) attended high schools, and fewer (2.6% vs. 11.0%, and 3.4% vs. 11.4%, respectively) schools of commerce, when parents had a university diploma, compared to lower educational levels. Similarly, fewer boys (0.0% vs. 12.3%, and 1.7% vs. 13.0%, respectively) attended schools of commerce, when parents had a university diploma; however, boys' attendance of high schools was unaffected by parental education.

## General Discussion

This study set out to investigate sex differences in general knowledge among high-school students in a more heterogeneous sample than previous research and by using a measure of general knowledge that was specifically tailored for German-speaking adolescents. Our study provides substantial evidence that the German GKT is a psychometrically sound and valid instrument. However, the higher-order structure proposed by [6] could not be fully replicated; second-order general knowledge was composed of four, not of six, first-order factors. Meta-analytical aggregation of previous studies suggested that sex differences were mostly heterogeneous and, across domains, of modest size at best. We obtained large sex differences in our data, but observed that general knowledge and its first-order factors were not independent of schooling and were moderated by parental education that also determined children's educational career. Sex differences in general knowledge could be explained to a large part in terms of selection processes, resulting in an overrepresentation of boys in school types where general knowledge was overall high, and an overrepresentation of girls in school types where general knowledge was overall low. Moreover, sex differences varied in size, dependent on school type, but also on individual schools. Broadly concordant with their specific curricula, increases in general knowledge and its first-order factors over time varied with school type. Controlling for these factors, sex differences were rendered small or even negligible, i.e., markedly less than 0.20*d* or insignificant.

Studies on school achievement [30], [31] and cross-national data on sex differences in school achievement [29] highlight that differences in schooling, parental education, and parental involvement are major determinants of school success. Contextual and sociocultural factors may act as barriers with regard to educational success, especially for girls [23]–[26]. In the present study, parental education affected children's general knowledge on multiple levels, determining children's educational career and disadvantaging specifically girls. Moreover, parental education moderated children's general knowledge via interactions with school type and individual schools that likely mirrored effects of parental involvement on children's school success. Being mostly confined to samples that stemmed from one and the same, or from few but overly similar, institutions, previous research likely underestimated the variability of general knowledge that can be attributed to differences in schooling, but also catchment area, that was observed here.

Remaining sex differences, regarding an advantage of boys over girls in general knowledge, were in size comparable to sex differences in general intelligence, fluid intelligence, and crystallized intelligence, i.e., small. Previous research highlighted the important role of especially Gc for knowledge acquisition [40]–[43]. Hence, the advantage of boys observed here is in good agreement with prior evidence.

Unexpectedly, we obtained unique evidence of a first-order factor covering the humanities, where girls excelled boys by a small margin. We propose to interpret this result in view of specific interests that appear to be more pronounced among girls. A preference of women for the arts and the humanities is well-known from research in higher education [44]. More generally, interests were also found to affect knowledge acquisition besides Gc (e.g., [43], [45]). However, the loading pattern in the humanities factor led to yet a further insight: positive knowledge in specific domains need not be accompanied by positive knowledge in other domains; i.e., governed by specific interests, various domains of general knowledge do not necessarily form a positive manifold (cf. [9]). In conclusion, the obtained results do not lend strong support to the interpretation that sex differences in general knowledge are driven by evolutionarily developed and genetically predisposed interests [6], [10], [17], [19]. Moreover, in the absence of biological data, such an interpretation must remain speculative at best. Instead, sex differences were found to be associated with selection processes, schooling, and parental education; knowledge increases over time were broadly concordant with the curricula of the different school types. The very pattern of sex differences interpreted by some authors as indicative of biologically predisposed interests could thus be explained in large part by effects of social norms, socialization, and schooling. Controlling for these effects, remaining sex differences were only small and consistent with prior evidence on sex differences in general intelligence, Gf and Gc. Differences in Gc, i.e., concerning verbal ability, language development, reading comprehension, and verbal reasoning, may be the driving factors of remaining sex differences in general knowledge; with regard to the humanities factor, were girls excelled boys, specific interests that also show no obvious biological roots or functions appear to be the driving factor.

The larger score variance of boys in general knowledge and most domains observed in the current study is consistent with prior evidence regarding general intelligence [22]. Remaining sex differences in general knowledge may thus partially stem from this greater male variability, whose roots may lie in an X-chromosomal pathway of inheritance of genes affecting the development of mental abilities [46]. We suggest investigating the greater male variability in general knowledge in more detail in future studies, utilizing and examining tail ratios, i.e., ratios of the proportions of boys and girls above a predefined cutoff, which may be beneficially used to directly assess and quantify sex differences in the spread of variability (see [47]).

Limitations of the present study pertain to its cross-sectional design which precludes strong conclusions regarding causal relationships; i.e., apparent gains in general knowledge over time could have been, partly or fully, caused by cohort effects. Likewise, selection processes were inferred from outcome, but were not assessed with regard to the admission of student to individual schools. Associations between parents' education and children's general knowledge were also likely confounded by genetic effects, i.e., parents with higher educational levels and their children may have had a better general knowledge because of the same set of genes that had been passed on. These effects could not be controlled for in the present study. General intelligence, Gf, Gc, and personality traits and learning styles, reportedly affecting general knowledge [8], [10]–[12], [14], [15], [28], [32], were not controlled in the present study, which may have influenced results. Finally, results of the present study are only valid with regard to the GKT and its variants. They may not generalize to other measures of general knowledge or domains of general knowledge that are not covered by the GKT.

In conclusion, the GKT was found a psychometrically sound and valid instrument that can therefore be recommended for use in further research. General knowledge was composed of four first-order factors, one of which referred to a specific interest in the humanities that was specifically pronounced among girls. Large naïve estimates of differences in general knowledge, favouring boys, could be explained to a large part by differences in schooling and selection processes that were moderated by parental education and specifically disadvantaged girls. Future research should focus of the effects of specific interests on the acquisition of general knowledge, controlling for general intelligence and Gc.

## Supporting Information

Checklist S1 **PRISMA checklist.**
(DOC)
